# C-type natriuretic peptide (CNP) in the paraventricular nucleus-mediated renal sympatho-inhibition

**DOI:** 10.3389/fphys.2023.1162699

**Published:** 2023-04-04

**Authors:** Hong Zheng, Tapan A. Patel, Xuefei Liu, Kaushik P. Patel

**Affiliations:** ^1^ Division of Basic Biomedical Sciences, Sanford School of Medicine of the University of South Dakota, Vermillion, SD, United States; ^2^ Department of Cellular and Integrative Physiology, University of Nebraska Medical Center, Omaha, NE, United States

**Keywords:** C-type natriuretic peptide, paraventricular nucleus, renal sympathetic nerve activity, volume reflex, central nervous system

## Abstract

Volume reflex produces sympatho-inhibition that is mediated by the hypothalamic paraventricular nucleus (PVN). However, the mechanisms for the sympatho-inhibitory role of the PVN and the neurochemical factors involved remain to be identified. In this study, we proposed C-type natriuretic peptide (CNP) as a potential mediator of this sympatho-inhibition within the PVN. Microinjection of CNP (1.0 μg) into the PVN significantly decreased renal sympathetic nerve activity (RSNA) (−25.8% ± 1.8% vs. −3.6% ± 1.5%), mean arterial pressure (−15.0 ± 1.9 vs. −0.1 ± 0.9 mmHg) and heart rate (−23.6 ± 3.5 vs. −0.3 ± 0.9 beats/min) compared with microinjection of vehicle. Picoinjection of CNP significantly decreased the basal discharge of extracellular single-unit recordings in 5/6 (83%) rostral ventrolateral medulla (RVLM)-projecting PVN neurons and in 6/13 (46%) of the neurons that were not antidromically activated from the RVLM. We also observed that natriuretic peptide receptor type C (NPR-C) was present on the RVLM projecting PVN neurons detected by dual-labeling with retrograde tracer. Prior NPR-C siRNA microinjection into the PVN significantly blunted the decrease in RSNA to CNP microinjections into the PVN. Volume expansion-mediated reduction in RSNA was significantly blunted by prior administration of NPR-C siRNA into the PVN. These results suggest a potential role for CNP within the PVN in regulating RSNA, specifically under physiological conditions of alterations in fluid balance.

## Introduction

Natriuretic peptide family contains three peptide hormones: atrial natriuretic peptide (ANP), brain natriuretic peptide (BNP), and C-type natriuretic peptide (CNP) (Sudoh et al., 1988; Brenner et al., 1990). CNP, as a vasoactive and anti-proliferative peptide, shares sequence homology and biological actions with the cardiac peptides ANP and BNP (Clavell et al., 1993; Horio et al., 2003). CNP mRNA has been identified in vascular endothelium (Stingo et al., 1992), cardiac, renal, skeletal (Vollmar et al., 1993) and reproductive tissues (Nielsen et al., 2008). Compared to ANP and BNP, the concentration of CNP is lower in plasma (Nielsen et al., 2005). However, in human cerebrospinal fluid (CSF) (Kaneko et al., 1993), the concentration of CNP is the highest among the natriuretic peptides. In addition, the content of immunoreactive CNP in ovine hypothalamic and pituitary extracts is approximately 50-fold higher than BNP and ANP (Imura et al., 1992; Herman et al., 1993). These distribution characteristics of CNP suggest CNP may play an essential role in the central nervous system. However, its role in central cardiovascular regulation and the underlying mechanisms still need to be better understood.

The hypothalamic paraventricular nucleus (PVN) integrates and responds to various neurohumoral signals regulating sympathetic activity and extracellular fluid volume (Pyner 2009). PVN is a critical site for the coordination and integration of neurogenic and humoral signals to affect the cardiovascular system and renal function in response to changes in blood volume (the volume reflex) in the body. The PVN includes neuroendocrine neurons that project to the median eminence, posterior pituitary, and pre-autonomic neurons that project to the brainstem and spinal cord regions (Swanson and Kuypers 1980; Dampney et al., 2005). The PVN projects to the rostral ventrolateral medulla (RVLM) (Shafton et al., 1998), which contributes to the changes of sympathetic activation. The PVN has also been well known for its role in the volume reflex, where a challenge of an increase in volume produces a reflex decrease in RSNA in different animal models, including dogs, sheep, rabbits, and rats (Haselton et al., 1994; Li et al., 2003; Coote 2005; Zheng et al., 2022). In the PVN, the parvocellular neurons which dictate renal sodium reabsorption by their actions *via* the renal nerves, are next to the magnocellular neurons that dictate water reabsorption. This adjacent location of these neuronal populations is ideal for regulating sodium and water, which contribute to the total extracellular fluid volume and blood volume in the body.

There are three types of natriuretic peptide receptors (NPR), type A (NPR-A), type B (NPR-B), and type C (NPR-C). It is known that NPR-C is highly expressed in the mammalian hypothalamus (Sumners and Tang 1992; Peng et al., 1996). CNP has been implicated in regulating the hypothalamic-pituitary-adrenal axis (Charles et al., 1995). Studies have shown that CNP augmented the hypothalamic-pituitary-adrenal axis at baseline and in response to hemorrhage (Charles et al., 1995). Intracerebroventricular infusions of CNP lower arterial pressure and plasma cortisol levels (Charles et al., 1992), and inhibit adrenocorticotropic hormone secretion (Guild and Cramb 1999). Furthermore, CNP has a inhibitory effect on L-type Ca^2+^ current and excitability in magnocellular neurosecretory cells that is mediated by the NPR-C (Rose et al., 2005).

Thus, it is imperative to determine whether CNP regulates sympathetic nerve activity in the PVN through the RVLM-projecting PVN neurons (PVN-RVLM neurons). The purpose of this study was to investigate 1) if microinjection of CNP inhibits basal renal sympathetic nerve activity (RSNA), 2) if this response is unique to RSNA or also observed in other sympathetic nerves like lumbar sympathetic nerves, 3) if picoinjection of CNP inhibits the basal discharge of the PVN-RVLM neurons, 4) if NPR-C are present on the PVN-RVLM neurons, 5) if renal sympatho-inhibition mediated by CNP from the PVN is blunted by gene silence of NPR-C within the PVN, and finally 6) if renal sympatho-inhibition mediated by acute volume expansion is blunted by gene silence of NPR-C within the PVN.

## Materials and methods

### Animals

Male Sprague-Dawley rats were used weighing between 250 and 300 g (SASCO Breeding Laboratories, Omaha, NE). Rats were housed with a 12-h light-dark cycle at ambient 22°C 30%–40% humidity. Laboratory chow and tap water were available *ad libitum*. The study was approved by the Institutional Animal Care and Use Committee of the University of Nebraska and was performed under the guidelines of the American Physiological Society and the National Institutes of Health Guide for the Care and Use of Laboratory Animals.

### General procedures

Rat was anesthetized by urethane (0.75 g/kg i.p.) and α-chloralose (70 mg/kg i.p.). Adequate depth of anesthesia was assessed by the absence of corneal reflexes and paw withdrawal response to a noxious pinch. Body temperature was maintained at 37°C with a temperature controller (ATC 1000, World Precision Instruments, Sarasota, FL). The right femoral artery and vein were cannulated for the recording of arterial blood pressure and administration of saline and drugs. The mean arterial pressure (MAP) and heart rate (HR) were recorded by a PowerLab data acquisition system (8SP, ADInstruments, Colorado Springs, CO).

### Renal and lumbar sympathetic nerve activity (LSNA) recordings

RSNA was recorded as previously described (Zheng et al., 2006). The renal nerve or lumber nerve was identified through a retroperitoneal incision. The distal end of the nerve was cut. The nerve was placed on a bipolar platinum electrode and insulated with gel (Wacker, St. Louis, MO). The signals were amplified with an AC/DC amplifier (A-M Systems, Carlsborg, WA) with a low-frequency cutoff at 60 Hz and a high-frequency cutoff at 3 kHz. The signals were integrated at a time constant of 10 ms. The background noise was determined after cutting the central end of the nerve at the end of the experiment and subtracted from the values of RSNA and LSNA. The raw RSNA, raw LSNA, integrated RSNA, integrated LSNA, arterial pressure, and HR were recorded on a PowerLab system. RSNA and LSNA were expressed as a percent change from the baseline value. The change in RSNA, LSNA, MAP, and HR from baseline to peak change was calculated for each microinjection experiment.

### Microinjections into the PVN

Rat was placed in a stereotaxic apparatus (David Kopf Instruments, Tujanga, CA). A longitudinal incision was made on the head. The bregma was exposed and a small burr hole was made. The coordinates for the right PVN, determined by the Paxinos and Watson atlas (Paxinos and Watson 1986), were 1.5 mm posterior to bregma, 0.4 mm lateral to the midline, and 7.8 mm ventral to the dura. A thin needle (0.2 mm OD) connected to a microsyringe (Hamilton, Reno, NV) was lowered into the PVN. Two doses of CNP (Sigma-Aldrich, St. Louis, MO) were injected into the PVN (0.5 and 1.0 μg, in a volume of 100 nL) randomly. In a separate group of rats, the vehicle control, 100 nL of artificial CSF (aCSF) was microinjected into the PVN. The effects on RSNA, LSNA, MAP and HR were monitored. At the end of the experiment, Chicago blue dye (100 nL, Sigma-Aldrich) was injected for histological identification.

### Extracellular single-unit recording *in vivo*

Rat was placed in a stereotaxic apparatus. The stereotaxic coordinates for the PVN were determined according to Paxinos & Watson’s atlas (Paxinos and Watson 1986). Three tracks were explored for extracellular recording, from −1.4 to −2.1 mm caudal to bregma, 0.4 mm lateral (right side) to the midline, and with a depth of 7.4–8.6 mm ventral to the dorsal surface. The extracellular single-unit recording was carried out by a single micropipette filled with 0.5 M sodium acetate containing pontamine sky blue. The spontaneous activity of neurons was amplified with an AC/DC amplifier (Model IX1, Dagan Corporation, Minneapolis, MN) with a low-frequency cutoff at 30 Hz and a high-frequency cutoff at 3 kHz. The neuronal discharge was recorded on a PowerLab system. The frequency of the neuronal discharge was analyzed with software (SpikeHistogram, ADInstruments). The pontamine sky blue was iontophoresed (−15 μA, 10 min) to mark the site of the recorded neuron, and other recording sites were extrapolated from the marked point according to Paxinos & Watson’s atlas.

### Identification of RVLM projecting PVN neurons

Antidromic stimulation was used to identify PVN-RVLM neurons *in vivo*. The RVLM (−12.5 mm caudal to the bregma, 2.1 mm right of the midline and 9.2 mm ventral to the surface of the cerebellum) was identified by a 15–20 mmHg blood pressure response to microinjection of NMDA (100 pmol) during a 10-s period (Lin et al., 2005). RVLM stimulation was ipsilateral to PVN recording. Subsequently, a concentric bipolar electrode (500 µm OD, tip tapered at 60°, World Precision Instruments) was placed at the same location in the RVLM to allow for antidromic stimulation with a stimulator (Model A310, World Precision Instrument).

When a spontaneously active neuron was recorded, standard tests were performed to ensure its antidromic nature (Lipski 1981). In brief, first we examined if the neurons responded to RVLM stimulation (pulse width: 3–10 ms; current intensity: 0.1–1 mA; stimulation frequency: 0.5 Hz) with consistent onset latency spikes at a discrete stimulus threshold. Second, we examined if the neurons responded to each pulse in a high-frequency 300 Hz stimulus train. The collision test was performed to examine whether the stimulus-evoked spikes can be canceled by spontaneous action potentials. A window discriminator (Model 74-60-1C, World Precision Instruments) and oscilloscope (Model OS-5100RA, LG, Korean) were used to discriminate the PVN spontaneous action potential, and the signal was translated to TTL pulse as a trigger with an adjustable latency to evoke the RVLM stimulation. At the end of the experiment, the stimulation sites in the RVLM were marked by passing 1 mA of anodal direct current for 20 s.

### Pressure picoinjection with extracellular single unit recording *in vivo*

To combine PVN single unit recording with pressure ejection, multi-barrel glass micropipettes (three barrels) were used. Single micropipettes were pulled for recording. Two ejection pipettes were pulled (Multi-pipette Puller PMP-107, MicroData Instrument, S. Plainfield, NJ) from thick-walled glass capillary tubing with a calibrated narrow inner diameter (O.D. = 1.0 mm, thick wall = 0.5 mm), which was redefined to a final outer diameter of approximately 30 μm. The recording and ejection pipettes were then assembled in a specially designed electrode holder (HMD-2, Narishige, Japan) to permit the tips and as long a part of the tapering ends as possible to touch one another (Akaoka et al., 1992). The tip of the recording pipette was advanced 30 μm beyond the ejection tip. About 5 nL volume of CNP (0.05 μg) was ejected by air pressure ejection (20 psi; pulse duration: 10–50 ms) (Picospritzer, General Valve Corporation, Fairfield, NJ), which was measured by microscope with a calibrated graticule. These parameters for picoinjection were based on the studies from others (Seagard et al., 1999; Wang et al., 2009). A vehicle (5 nL aCSF) picoinjection to control for volume effect were performed. The discharge rates of PVN neurons were averaged during two time periods; 1) 60 s for baseline discharge, 2) 20 s, immediately after CNP or aCSF picoinjections. The PVN neurons were considered to be responsive if their peak discharge frequency after treatments was changed by at least 30% above the baseline.

### Histology of recording sites within the PVN

At the end of the experiment, the rat was euthanized with an overdose of anesthesia. The rat brains were then removed, frozen, and sectioned. The dye spots for recording sites in the PVN and the sites of electrolytic lesion in the RVLM were identified with a light microscope. Rats with recording sites within the PVN were used for data analysis. The location of the dye spot was transferred to a histological map based on the rat atlas Paxinos & Watson’s atlas.

### Retrograde tract tracing combined with immunofluorescent staining

The fluorescent tracer latexgreen (4%, 100 nL, ThermoFisher) was injected bilaterally into the region of the RVLM in three rats 7 days prior to sacrifice. This allowed the tracer enough time to be transported from the RVLM to the neurons that project to the PVN (Li et al., 2002; Llewellyn et al., 2012). After sacrifice, the brain was removed and postfixed at 4°C for 4 h in 4% paraformaldehyde solution and placed in 20% sucrose. The brain was blocked in the coronal plane, and sections 30 μm in thickness were cut with a cryostat. The sections were incubated with 10% donkey serum in phosphate-buffered saline (PBS) for 1 h and were then incubated with a primary antibody against NPR-C (rabbit monoclonal antibody, 1:500, Abcam, Cambridge, MA) overnight at 4°C. After being washed with PBS, the sections were incubated with Cy3-conjugated donkey anti-rabbit secondary antibody (1:400, Jackson ImmunoResearch, West Grove, PA) for 2 h. The nuclei were stained by 4′,6-diamidino-2-phenylindole (DAPI). The sections were coverslipped with fluoromounting-G (Southern Biotech, Birmingham, AL). Distribution of NPR-C by immunofluorescence and latexgreen within the PVN was viewed using an Olympus fluorescence microscope equipped with a digital camera (Qimaging, Canada).

### Validation of knockdown of NPR-C using siRNA *in vitro*

siRNA targeting NPR-C (3 types of siRNA, siRNAa, siRNAb, siRNAc) and a negative control siRNA (scRNA), were purchased from ThermoFisher Scientific. Transient transfection was performed in NG108 cells using lipofectamine 2,000 as per the manufacturer’s instructions. Changes in NPR-C protein expression were analyzed by Western blot analysis after 24 h of transfection.

Protein samples were mixed with an equal volume of 2% × 4% SDS sample buffer. The sample was then loaded onto a 7.5% SDS-PAGE gel for electrophoresis at 40 mA/each gel for 60 min. The fractionated proteins on the gel were electrophoretically transferred onto the PVDF membrane at 300 mA for 90 min. The membrane was incubated with 5% milk-Tris-buffered saline with Tween 20 solution for 30 min. The membrane was incubated with primary antibody (anti-rabbit NPR-C, 1:1000, Abcam; anti-rabbit Glyceraldehyde-3-Phosphate Dehydrogenase-GAPDH, 1:1000, Santa Cruz Biotechnology) at 4°C overnight. After washing, the membrane was incubated with a secondary antibody (goat anti-rabbit IgG, peroxidase conjugated, 1:5000, PIERCE, IL) for 40 min. The signals were visualized using an enhanced chemiluminescence substrate (Thermo Scientific) and detected by UVP digital image system (UVP LLC, Upland, CA).

### Validation of knockdown of NPR-C using siRNA *in vivo*

siRNA targeting NPR-C (using siRNAb) or a negative control siRNA (scramble, scRNA group) was microinjected into the PVN (unilateral injection) under anesthetic conditions. The experiments were performed at 3–4 h after siRNAb treatment in the two groups of rats. The high dose of CNP was injected into the PVN (1.0 μg, in a volume of 100 nL on the same side). The effects on RSNA, MAP and HR were monitored. At the end of the experiment, 100 nL of Chicago blue dye was injected into each microinjection site for later histological identification.

### Volume expansion and change in RSNA after NPR-C knockdown in the PVN

Continuous perfusion of 0.9% NaCl solution *via* the catheter in the right femoral vein was used as the volume expansion stimulus. The total perfusion volume was 10% body weight, which was infused at a constant flow rate over a 40-min period. To determine the effect of blockade of NPR-C in the PVN on volume expansion-induced changes in RSNA, rats were treated with either siRNAb or aCSF (200 nL) into the PVN (bilateral injection). 200 pmol of siRNAb in 200 nL were injected into the PVN over a 2-min period at 3–4 h before volume expansion. After injection of siRNAb or aCSF, RSNA, MAP, HR, and central venous pressure were consecutively monitored before (baseline) and during volume expansion. Central venous pressure was monitored *via* a cannula advanced to the junction of superior vena cava and right atrium *via* the carotid vein. The volume expansion produced a progressive increase in central venous pressure. A relationship between central venous pressure and RSNA changes was quantified and plotted.

### Statistical analysis

Data are presented as mean ± SEM. Analysis of RSNA, MAP and HR was performed by two-way repeated measures ANOVA for multiple comparisons. All other results were analyzed by one-way ANOVA followed by Bonferroni’s analysis for individual comparisons using Prism 7; GraphPad Software. *p* < 0.05 was considered statistically significant.

## Results

### Microinjection of CNP into the PVN on RSNA, LSNA, MAP, and HR

**FIGURE 1:**
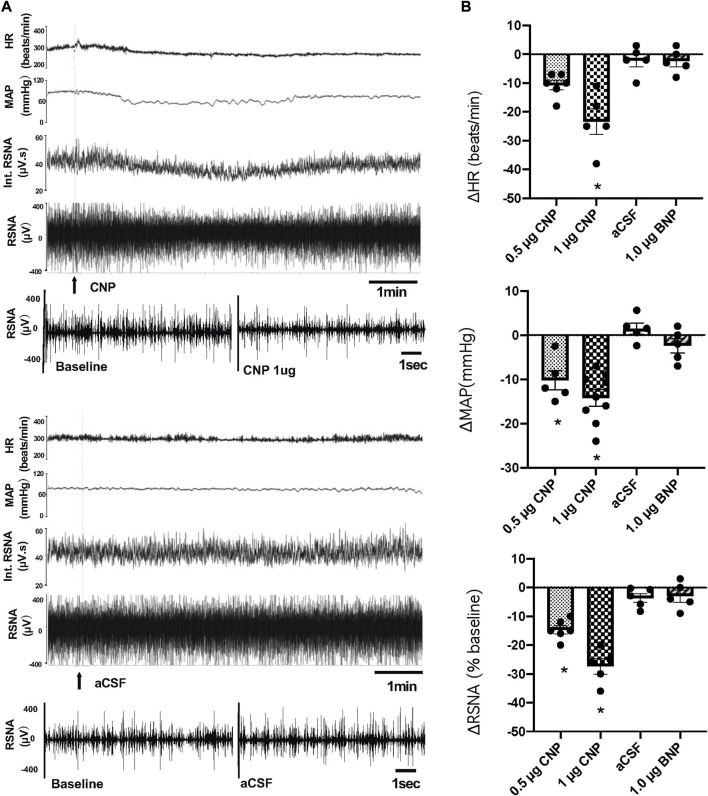
**(A)** Segments of original recordings demonstrating the representative responses of heart rate (HR), mean arterial pressure (MAP), integrated (Int) renal sympathetic nerve activity (RSNA), and raw RSNA to microinjections of C-type natriuretic peptide (CNP) (1.0 μg) or artificial cerebrospinal fluid (aCSF) (arrows) into the paraventricular nucleus (PVN). Bar = 1 min. **(B)** HR, MAP, and RSNA responses to the low dose of CNP (0.5 μg), high dose of CNP (1.0 μg), B-type natriuretic peptide (BNP) (1.0 μg) and aCSF microinjection into the PVN, n = 5–9. Values are presented as mean ± SE. *p < 0.05 compared with aCSF.

Microinjection of CNP in the PVN produced a dose-dependent decrease in RSNA, MAP and HR. The higher dose of CNP (1.0 μg) into the PVN significantly decreased RSNA (−25.8 ± 1.8 n = 5 vs. −3.6% ± 1.5% n = 5, *p* < 0.05), MAP (−15.0 ± 1.9 n = 9 vs. −0.1 ± 0.9 mmHg n = 5, *p* < 0.05) and HR (−23.6 ± 3.5 n = 5 vs. −0.3 ± 0.9 beats/min n = 5, *p* < 0.05) compared with microinjection of aCSF (Figure 1). A segment of original recordings demonstrating the representative responses to the higher dose of CNP microinjection is shown in Figure 1A. Microinjection of a lower dose of CNP (0.5 μg, n = 5) also significantly decreased RSNA and MAP, but not HR compared with microinjection aCSF. However, microinjection of BNP (1.0 μg, n = 5) did not significantly change RSNA, MAP or HR compared to microinjection aCSF (Figure 1B). Microinjections of CNP that missed the PVN (outside the boundary of the PVN) but were adjacent to the PVN (n = 3) had no significant effect on RSNA, MAP or HR (data not shown).

**FIGURE 2:**
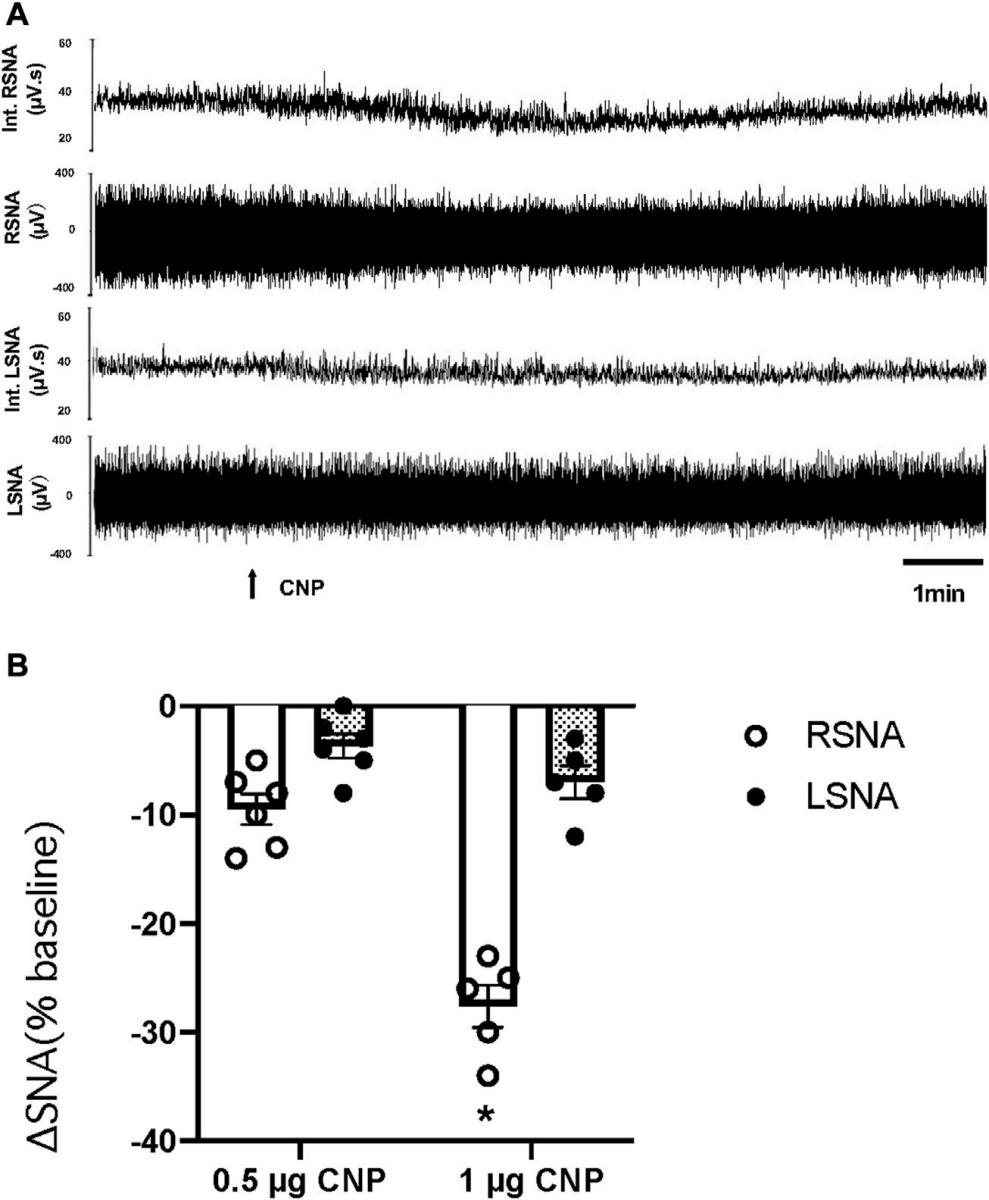
**(A)** Segments of original recordings demonstrating the representative responses of Int.RSNA, RSNA, integrated lumbar sympathetic nerve activity (int.LSNA), and LSNA to microinjections of CNP (1.0 μg) into the PVN. Bar = 1 min. **(B)** Cumulative data of change in RSNA and LSNA in response to two doses of CNP (0.5 and 1.0 μg) (n = 6) microinjected into the PVN. Values are presented as mean ± SE. *p < 0.05 compared with LSNA.

An additional set of experiments was performed to determine whether there was a decrease in LSNA comparable to that observed in RSNA to microinjection of CNP in the PVN. The LSNA and RSNA were recorded simultaneously during the administration of CNP in the PVN. When the changes in RSNA and LSNA were compared, RSNA decreased significantly more than LSNA after microinjection of the higher dose CNP (1.0 μg) in the PVN (Figure 2).

### Effect of CNP on RVLM projecting PVN neurons

**FIGURE 3:**
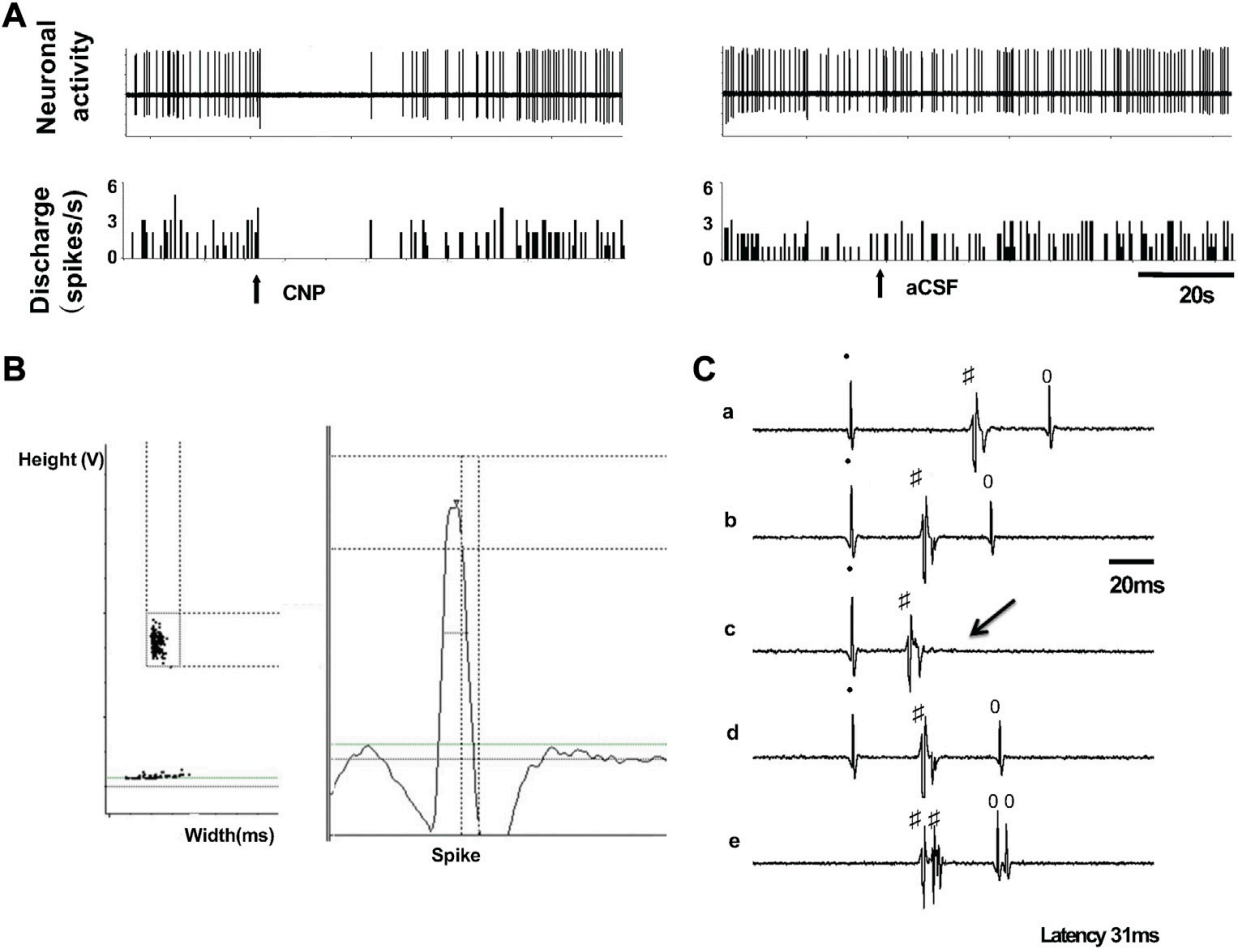
**(A)** Segments of original recordings from an individual PVN-RVLM neurons after picoinjection of CNP (right), or aCSF (left) in a rat. **(B)** Spike discriminator demonstrating a single-unit discharge. **(C)** Antidromic stimulation evoked action potential with constant latency (32 ms, a, b, and d), for the same neuron as in c, RVLM stimulation evoked an antidromic spike that was canceled (arrow) when the interval between spontaneous action potential and stimulation was reduced to <31 ms, and high frequency (333 Hz, 3 ms) following test (e). All segments (a through e) represent 2 superimposed sweeps.

Nineteen spontaneously active neurons were recorded in the PVN of normal rats with extracellular single-unit recording *in vivo*, of which 6 units were antidromically activated from the PVN-RVLM neurons (Figure 3). Each of the units was tested to identify those that were antidromically activated from the RVLM. First, the PVN-RVLM neurons responded to RVLM stimulation with consistent onset latency (39.1 ± 9.3 ms, average latency). Second, the PVN-RVLM neurons responded to each pulse in a high-frequency 300 Hz stimulus train. Then, we performed a collision test in the PVN-RVLM neurons. Figure 3C shows one neuron that had antidromic spikes evoked by stimulation of the RVLM with constant latency (31 ms), and the antidromic spike was canceled when the interval between the spontaneous action potential and the stimulation was reduced to <31 ms.

**FIGURE 4:**
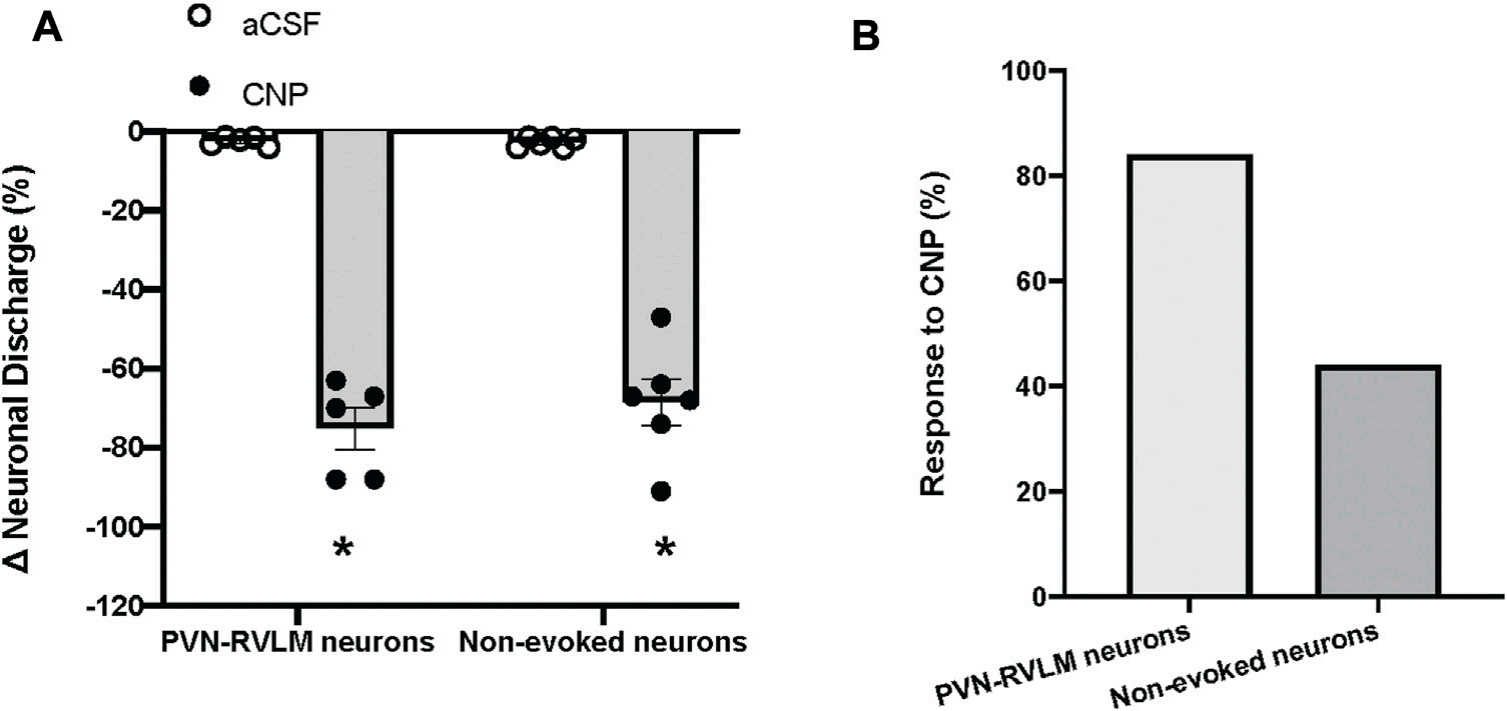
**(A)** Percent changes in discharge after picoinjection of CNP or aCSF on PVN-RVLM neurons (n = 5) or no-evoked neurons (n = 13), which did not evoke antidromic spikes by antidromic stimulation of the RVLM with constant latency. **(B)** Percent of PVN-RVLM neurons and no-evoked neurons response to CNP picoinjection. Values are presented as mean ± SE. *p < 0.05 vs. aCSF injected rats.

The baseline discharge rate of the PVN neurons was (2.8 ± 0.5 spike/s, n = 19). Due to the large variability of baseline neuronal discharge rate, the percentage of change was used for comparison before and after treatments. Picoinjection of CNP (0.05 μg, in 5 nL) significantly decreased the basal discharge in 5/6 PVN-RVLM neurons (76% ± 5% vs. 2% ± 6% n = 5, *p* < 0.05), and in 6/13 neurons (69% ± 6% vs. 2% ± 3% n = 6, *p* < 0.05) that were not antidromically activated from the RVLM, compared with picoinjection of aCSF (5 nL) (Figure 4). The majority of PVN-RVLM neurons (80%) were responsive to CNP compared to non-evoked neurons (40%). There were no significant changes in MAP or HR after picoinjection of CNP or aCSF within the PVN.

### Retrograde tracer combined with NPR-C immunofluorescent staining

**FIGURE 5:**
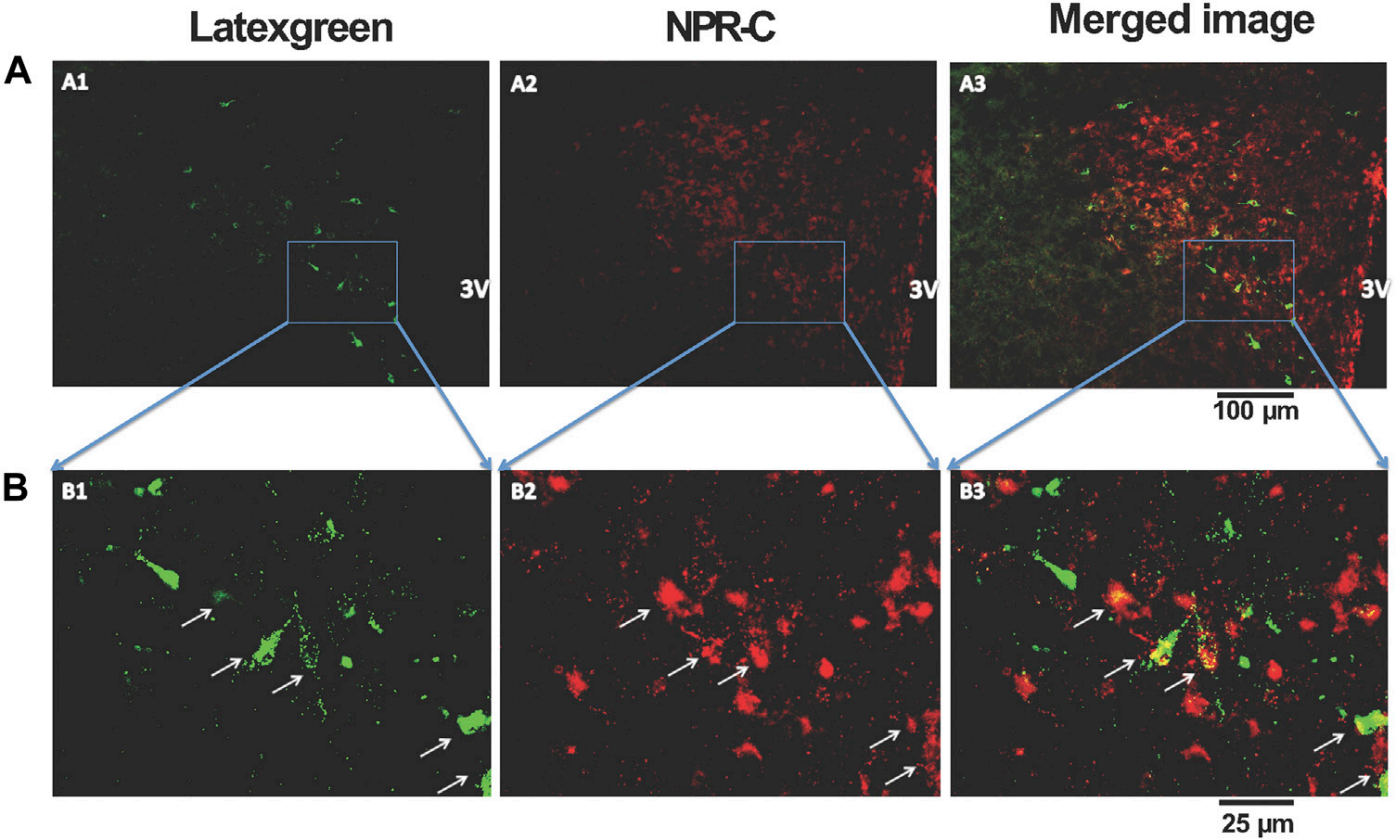
Representative low-magnification **(A)** and high-magnification **(B)** confocal images showing co-localization of immunoreactivity of retrogradely labeled PVN-RVLM neurons (A1 and B1, green) and NPR-C receptors (A2 and B2, red). Note that some retrogradely labeled neurons and NPR-C immunoreactivity are co-localized in the PVN (A3 and B3, white arrow). Scale bar, 100 μm **(A)** and 25 μm **(B)**. All images are single confocal optical sections. 3V, third ventricle.

To determine the spatial relationship between the NPR-C and the PVN-RVLM neurons, the retrogradely transported tracer latex-green was injected into the RVLM. These images illustrate neurons originating in the PVN project to the RVLM. Further, NPR-C receptors are present on PVN neurons that projected to the RVLM as detected by immunohistochemistry. Double-labeled neurons were mainly localized in the parvocellular portion of the PVN (Figure 5).

### Effect of knockdown of NPR-C using siRNA in NG108 cells

**FIGURE 6:**
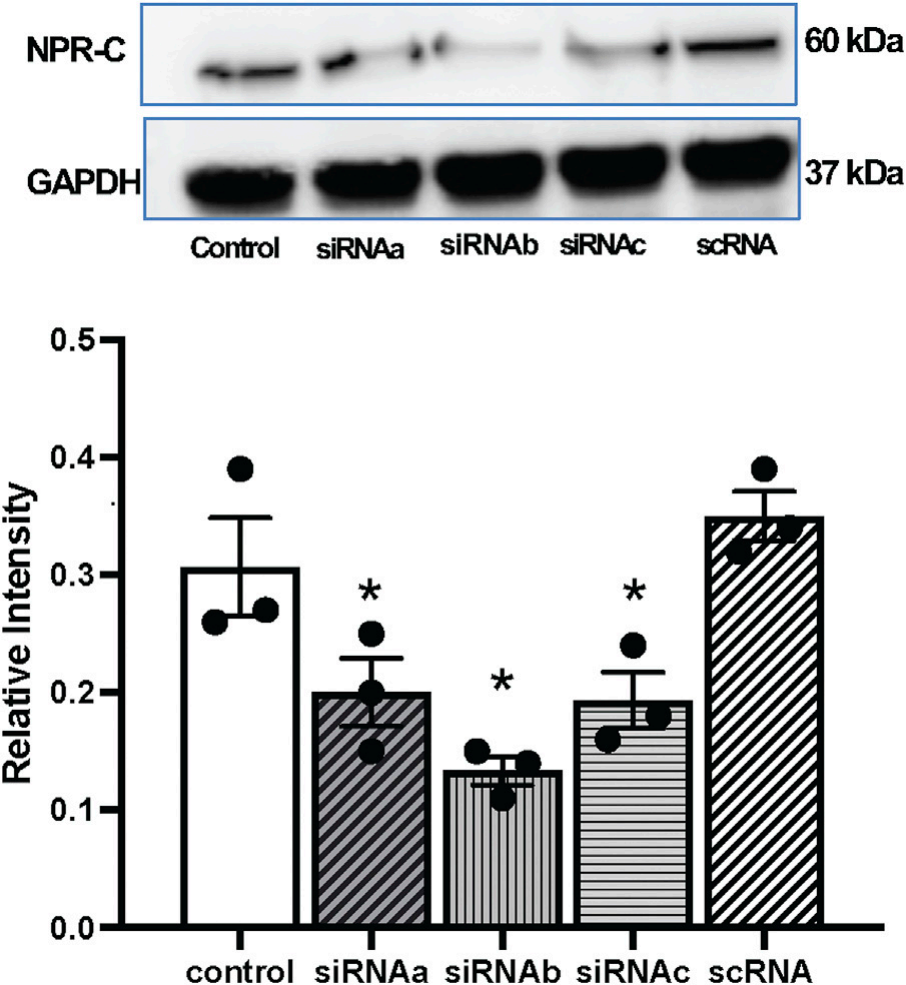
Representative western blots and mean values of NPR-C protein expression with NPR-C siRNA (siRNAa, siRNAb, siRNAc) and scramble RNA (scRNA) in neuronal NG108 cells. Values are presented as mean ± SE, n = 3. *p < 0.05 compared with the control group.

Three types of NPR-C siRNA mediated gene silencing of NPR-C were attempted *in-vitro*, utilizing NG108 cells and lipofectamine (Figure 6). These results indicated that NPR-C siRNA (siRNAa, siRNAb and siRNAc) are effective in blocking NPR-C receptors *in vitro*. NPR-C siRNAb was most effective in blocking NPR-C protein in NG 108 cells and was subsequently used for *in-vivo* silencing NPR-C within the PVN.

### Effect of pretreatment NPR-C siRNA into the PVN on CNP-induced inhibition in RSNA, MAP, and HR

**FIGURE 7:**
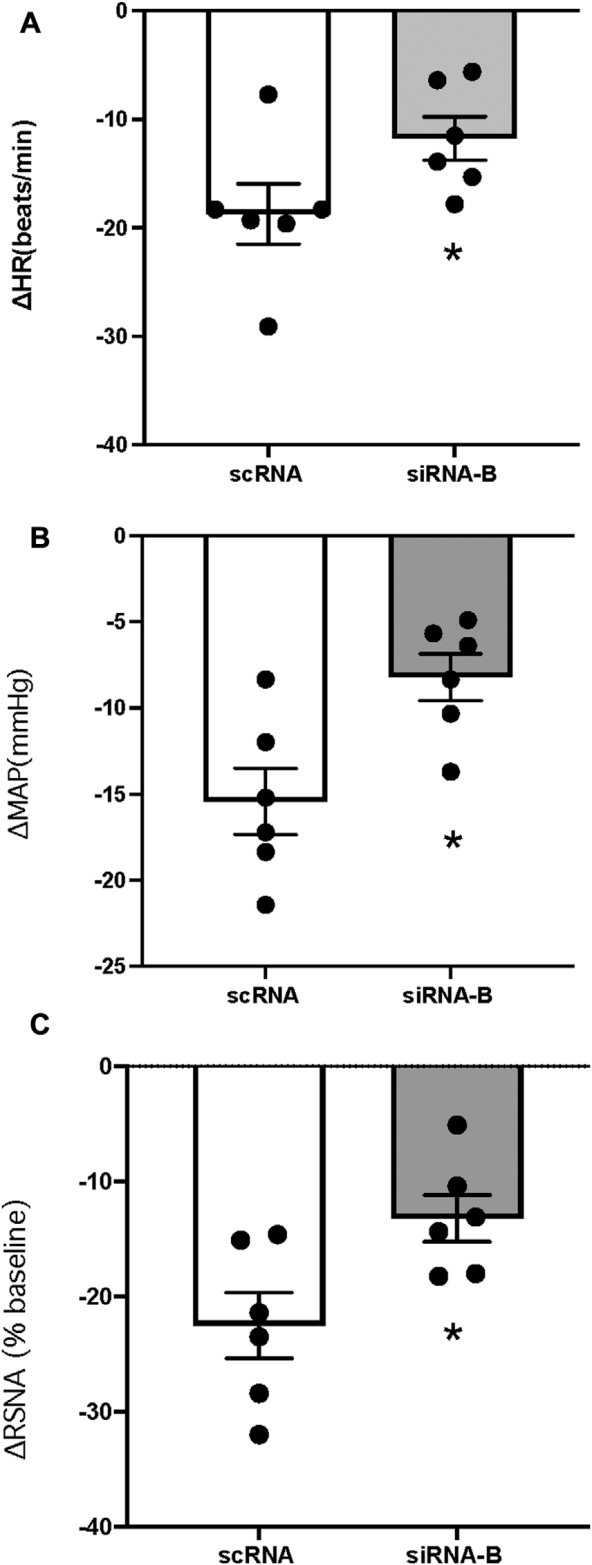
Change in HR **(A)**, MAP **(B)** and RSNA **(C)** in response to CNP (1.0 μg) (n = 6) microinjected into the PVN in two groups of rats that had prior microinjection of NPRC siRNAb or scRNA. Values are presented as mean ± SE. *p < 0.05 compared with scRNA group.

CNP (1 μg) was microinjected following (3–4 h) administration of scRNA or siRNAb (200 nL) for NPR-C into the PVN (unilateral injection) in rats. CNP induced inhibition of RSNA and decreased in MAP and HR in both groups of rats (Figure 7). Changes in RSNA, MAP and HR were attenuated by pretreatment with NPR-C siRNAb compared to scRNA in the PVN. These results indicated that NPR-C siRNA was effective in blocking CNP actions on NPR-C receptors *in vivo*. There were no significant differences in basal RSNA (39.5 ± 2.6 vs. 40.9 ± 3.6 μV s), MAP (85 ± 3 vs. 88 ± 4 mmHg) and HR (310 ± 12 vs. 328 ± 18 bpm, *p* > 0.05) between the siRNAb and scRNA groups.

### Effect of NPR-C siRNA on the volume expansion-induced RSNA inhibition

**FIGURE 8:**
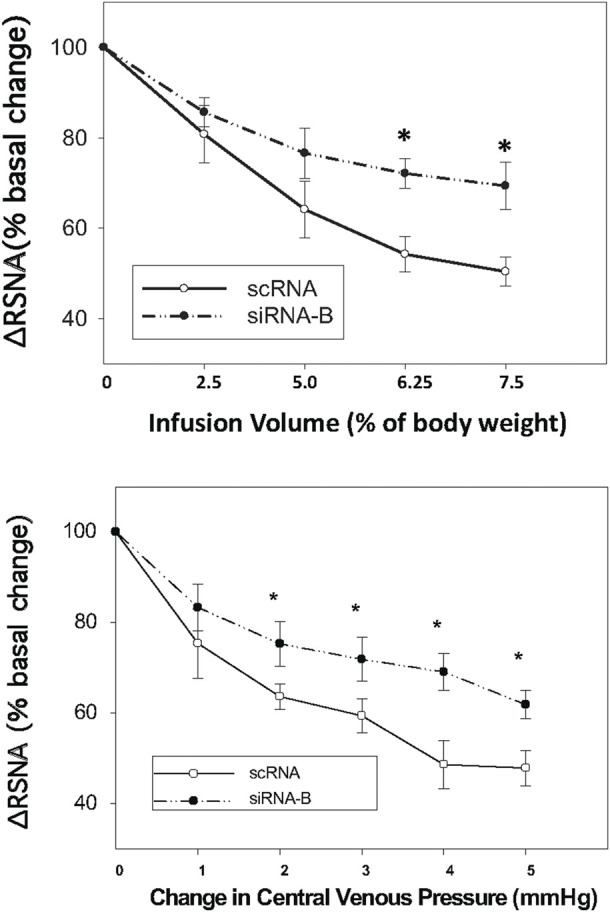
Infusion volume and central venous pressure (CVP)-mediated changes in RSNA in rats that had prior microinjections of NPR-C siRNAb or scRNA bilaterally within the PVN. Values are presented as mean ± SE. *p < 0.05 compared with scRNA group.

**FIGURE 9:**
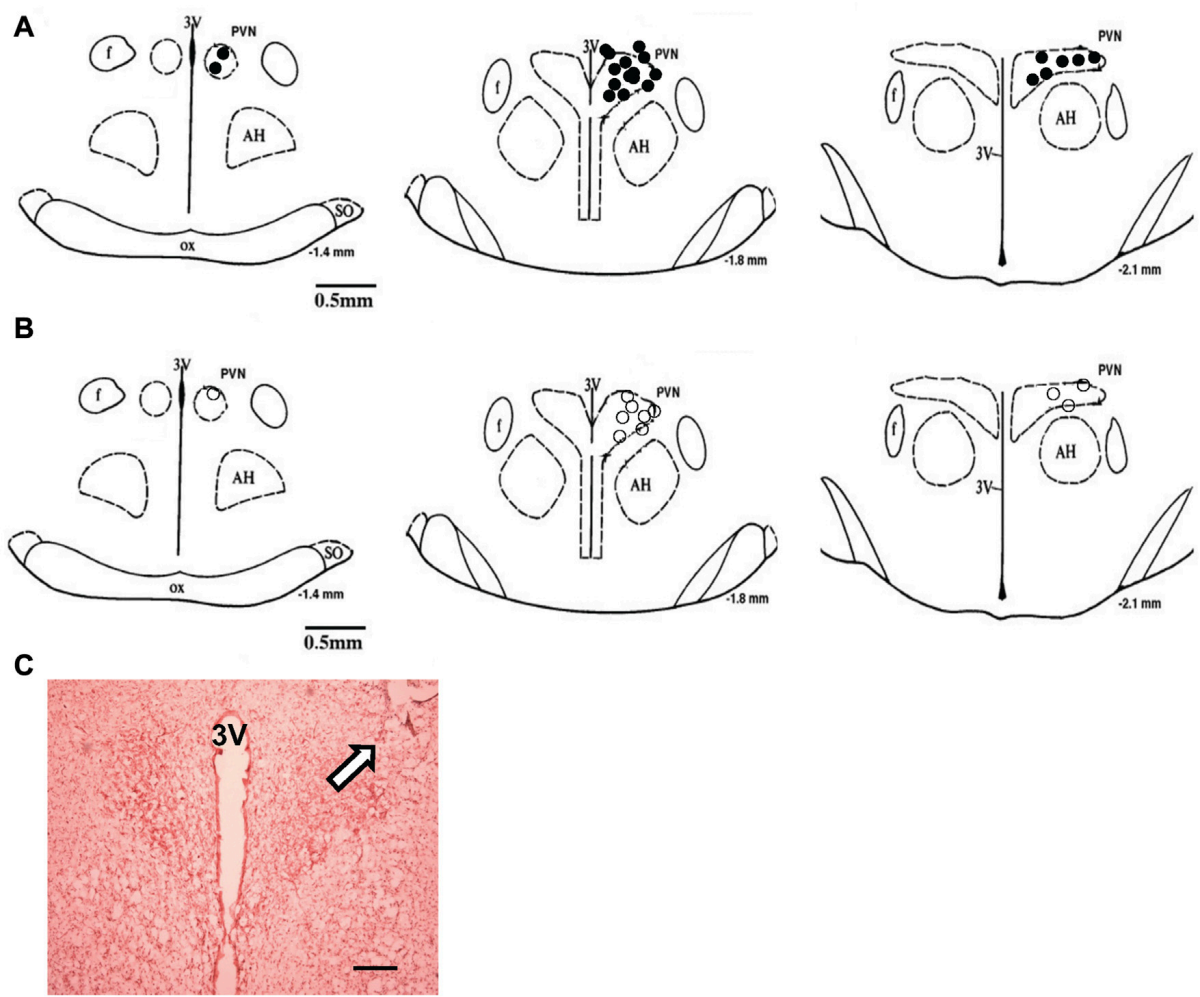
**(A)** The microinjection sites of CNP in the PVN to record RSNA or LSNA. **(B)** Approximate locations of the evoked and non-evoked PVN neurons stimulated by CNP. The distance (in mm) posterior to bregma is shown for each section. **(C)** Histological photo showing the microinjection site in the PVN of one rat. Arrowhead shows a marked injection site. Bar = 200 μm. AH, anterior hypothalamic nucleus; f, fornix; 3V, third ventricle; OX, optic tract; SO, supraoptic nucleus.

Administration of siRNAb for NPR-C into the PVN significantly attenuates the RSNA inhibitory response to acute volume expansion and resultant increase in central venous pressure (CVP) (Figure 8). While the negative control, prior scRNA micro-injected within the PVN did not show an attenuated response of RSNA inhibition induced by acute volume expansion. Figure 9 shows a schematic diagram illustrating the locations of microinjections of CNP within the PVN.

## Discussion

In the present study, we first observed that microinjection of CNP into the PVN significantly decreased RSNA, MAP and HR compared with microinjection of aCSF, or BNP. Second, microinjection of CNP into the PVN differentially affected RSNA more than LSNA. Third, picoinjection of CNP significantly reduced the basal discharge of PVN-RVLM neurons. Fourth, NPR-C are present on the PVN-RVLM neurons. Fifth, blocking NPR-C within the PVN attenuates the renal sympatho-inhibitory response to CNP microinjected into the PVN. Finally, blocking NPR-C within the PVN attenuates the renal sympatho-inhibitory response to acute volume expansion *in vivo*. These results are the first to systematically document a potential inhibitory role for CNP within the PVN in regulating RSNA under physiological conditions of changing blood volume.

The PVN has been recognized for a long time as a key region of the hypothalamus for neuroendocrine, autonomic, and behavioral homeostatic responses (Swanson and Sawchenko 1980). In terms of fluid balance, an acute volume expansion produces a reflex decrease in RSNA that is mediated by the PVN (Haselton et al., 1994; Li et al., 2003; Coote 2005; Zheng et al., 2022). However, the mechanism/s or possible neurotransmitter mediator/s for the sympatho-inhibitory action of the PVN remain to be identified. It has been reported that there is approximately 50-fold higher concentration of CNP compared to BNP and ANP in hypothalamic extracts (Imura et al., 1992; Herman et al., 1993), suggesting a possible contributory role for CNP in the hypothalamus. Specifically, previous studies have also reported a very high expression of CNP mRNA in the PVN (Herman et al., 1993). Consistent with these observations, in the present study we found that microinjection of CNP into the PVN significantly decreased RSNA, MAP and HR. Microinjection of BNP had minimal effects compared to those observed with CNP, consistent with the potential effects of high levels of CNP compared with BNP observed previously in the hypothalamus. The effect of CNP was specific for the PVN since microinjection of the drug in areas outside but adjacent to the PVN did not affect the RSNA and MAP. It is important to note that the renal sympatho-inhibition (decrease in RSNA) was significantly greater than that observed in the LSNA. The finding suggests that CNP may play an inhibitor role in the PVN regulating RSNA, specifically. These observations are consistent with the other studies demonstrating differential regulation of lumbar and renal sympathetic outflow from the PVN (Stocker et al., 2005).

It is of interest to note that inhibitory effect on RSNA originating in the PVN is not particularly new. Deering at el. showed that low amounts of the neuronal excitant DL-homocysteic acid preferentially evoke excitation of cardiac sympathetic activity and simultaneously evoke inhibition of renal sympathetic activity from some parvocellular regions of the PVN (Deering and Coote 2000). Paraventricular neurons elicit a volume expansion-like change of activity in sympathetic nerves to heart and kidney in the rabbit. Lovick et al. demonstrated that selectively destroyed more than 80% of parvocellular portion of the PVN resulted in abolishing of renal vascular response to systemic volume load (Lovick et al., 1993). Renal vasodilatation in response to acute volume load is attenuated following lesions of parvocellular neurons in the PVN in rats. Haselton et al. showed that a reflex reduction in renal sympathetic nerve traffic in response to plasma volume expansion was markedly attenuated following destruction of PVN parvocellular neurons (Haselton et al., 1994). These data suggest that parvocellular neurons of the PVN are involved in the reduction in renal nerve discharge during isotonic volume expansion. Pyner et al. also demonstrated that the activation of parvocellular neurons of the PVN in the volume reflex initiated by right atrial stretch (Pyner et al., 2002). They demonstrated that right atrial stretch induces renal nerve inhibition with concomitant increase in c-Fos expression in parvocellular neurons of the PVN in rats. Based on this evidence our observation showing inhibition of pre-autonomic neurons that influence RSNA to the kidneys by CNP in the PVN is consistent.

Although, we observed differential responses in RSNA versus LSNA to microinjection of CNP in the PVN, it is unclear whether this differential regulation of these nerves results from an action on separate neuronal populations within the PVN linked to specific autonomic neurons projecting differentially to renal versus lumbar sympathetic nerves. These results also provide corollary evidence to a previous study that showed that microinjection of CNP into the third ventricle of the rat brain potently inhibited the release of vasopressin (Shirakami et al., 1993; Yamamoto et al., 1997), which has an essential role in regulating blood volume and fluid homeostasis. Congruent with these observations, we observed that the knockdown of NPR-C with siRNA within the PVN blunted the acute volume expansion-induced renal sympatho-inhibition. These findings indicate that CNP in the PVN may play an important role in regulating renal nerves and consequently influence renal function involved in fluid balance. However, the details of this role in the pathophysiological conditions with altered regulation of blood volume and fluid homeostasis remain to be examined.

Using the method of extracellular single-unit recording *in vivo*, we found that picoinjection of CNP significantly decreased the basal discharge in 80% of PVN-RVLM neurons, compared with only 40% of the neurons that were not antidromically activated from the RVLM. It is well known that the PVN contains distinct subdivisions of large magnocellular neurons that project to the posterior pituitary, and small parvocellular neurons that project to preautonomic neurons in the RVLM and the intermediolateral nucleus of the spinal cord (Pyner 2009). The large magnocellular neurons project to the posterior pituitary and the supraoptic nucleus, are responsible for the release of vasopressin and oxytocin into the bloodstream. In contrast, the smaller parvocellular neurons project to preautonomic neurons in the RVLM and spinal cord. They activate the sympathetic nervous system and regulate cardiovascular function (Pyner 2009). One previous study showed that central CNP augmented the hypothalamic-pituitary-adrenal axis at baseline and in response to hemorrhage in conscious sheep (Charles et al., 1995). Furthermore, CNP has a potent and selective inhibitory effect on L-type Ca^2+^ current and on excitability in magnocellular neurosecretory cells in the PVN that is mediated by the NPR-C (Rose et al., 2005). Since CNP was also influential in increasing discharge in non-evoked neurons in the PVN, it is plausible that these particular neurons were involved in regulating neuroendocrine functions engaged in fluid and electrolyte homeostasis, independent of effects *via* the renal nerves.

To investigate the underlying link of CNP-mediated mechanisms in the neural pathways that regulate sympathetic nerve activity from the PVN, we employed retrograde tract tracing combined with immunofluorescent staining. The results showed NPR-C receptors are present on PVN neurons that projected to the RVLM. These double-labeled neurons were mainly localized to the parvocellular portion of the PVN. The antibody used to detect NPR-C is well characterized (Abdelalim et al., 2008). Therefore, it is unlikely to show false positives. CNP shows a high affinity for two receptors that have been denoted, NPR-B and NPR-C (Levin et al., 1998). NPR-C is commonly thought to act as a clearance receptor and sequester released natriuretic peptides, reducing local concentrations of the peptide available for cyclic guanosine monophosphate (cGMP) generation (Maack et al., 1987). However, recent evidence suggests that NPR-C may also interact with the adenylate cyclase signal transduction system, inhibiting cyclic adenosine monophosphate (cAMP) production and activating phosphodiesterase C without affecting cGMP levels (Anand-Srivastava et al., 1990; Murthy et al., 2000). Several effects of CNP appear to be mediated by the NPR-C signaling pathway. CNP/NPR-C signaling in the heart has been linked to electrophysiological activities. In cardiac myocytes, including the sinoatrial node myocytes, NPR-C mediates the CNP-induced inhibition of L-type Ca^2+^ current (Rose and Giles 2008). CNP has been also shown to exert an inhibitory effect on evoked neurotransmitter efflux through NPR-C binding (Trachte 2000). The activation of NPR-C alters adenylyl cyclase and cAMP levels *via* the activation of a pertussis toxin-sensitive Gi protein (Rose and Giles 2008). Previous studies have also shown that NPR-C is prominently expressed in the mammalian hypothalamus (Sumners and Tang 1992; Peng et al., 1996). Our data provide the first anatomical description for the expressing NPR-C on PVN-RVLM neurons, potentially important in blood volume regulation.

**FIGURE 10:**
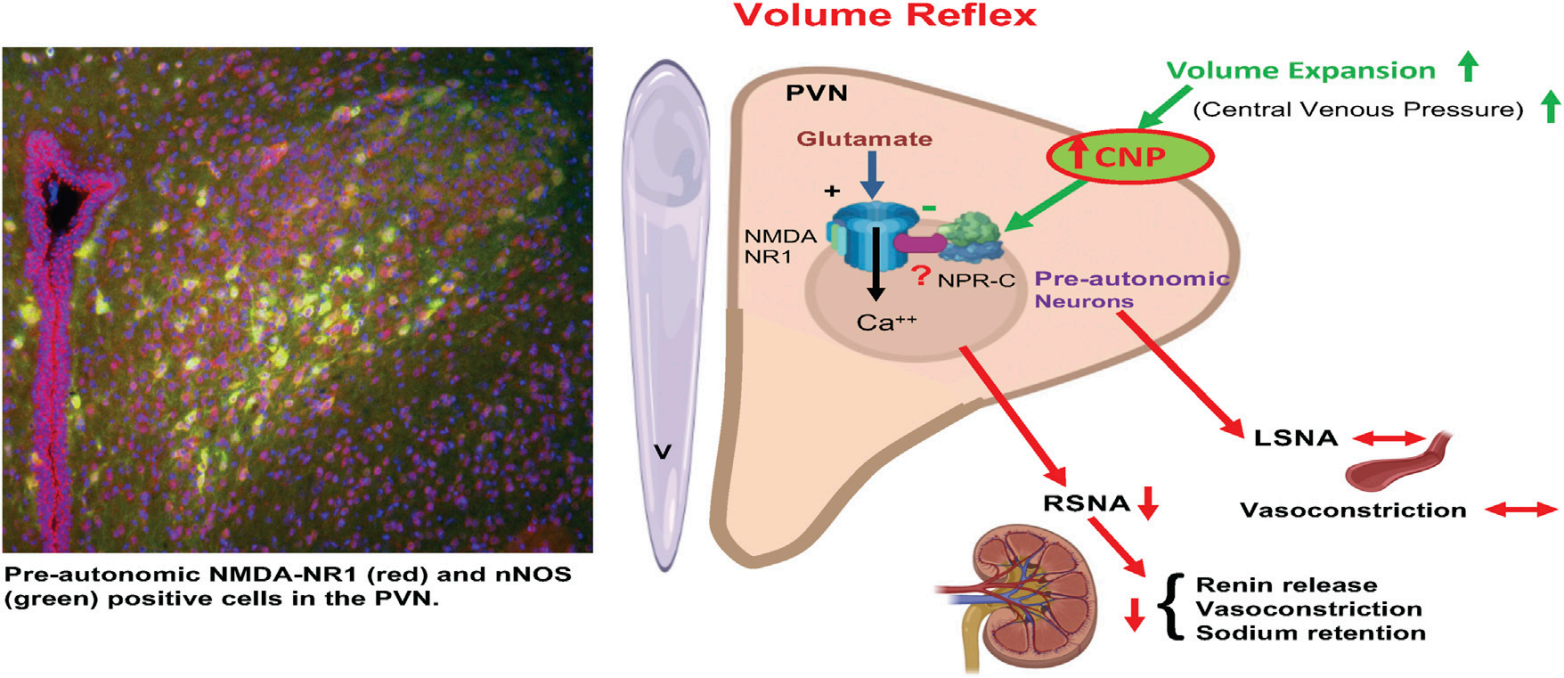
A schematic diagram is showing that activation of afferents from volume receptors in the low pressure side of the circulation due to volume expansion causes an increase in CNP in the PVN resulting in specific inhibition of preautonomic glutamatergic neurons (NR1 receptors) innervating the kidneys (RSNA) but not LSNA innervating other general vasculature. Inhibition of RSNA would cause a decrease in renin release, reduced renal vasoconstriction and reduced sodium retention by renal tubules. CNP in the PVN inhibits pre-autonomic neurons that project to the kidneys by acting on the NPR-C. (Templates used from BioRender).

It is well accepted that baseline sympathetic outflow mediated by the RVLM is mainly dependent on the spontaneous activity of preautonomic neurons in the RVLM (Guyenet et al., 1989; Dampney 1994; Pyner and Coote 1999; Pyner and Coote 2000). Our results imply that NPR-C may be crucially located on PVN-RVLM neurons such as to be involved in regulating sympathetic outflow. The results show that CNP may play an inhibitor role on PVN-RVLM neurons by binding to NPR-C, although the participation of NPR-B could not be excluded. The pattern of discharge activity of PVN-RVLM neurons directly influences the sympathetic never activity (Chen et al., 2010). We have shown that the microinjection of CNP into the PVN significantly inhibits RSNA with a concomitant reduction in neuronal firing of PVN-RVLM neurons. Thus, this change in the excitability of RSNA could account for the ability of CNP within the PVN to inhibit the firing of the PVN-RVLM neurons. The exact mechanism by which the actions of CNP inhibits the preautonomic glutamatergic neurons remains to be explored. One intriguing possibility is that CNP may be increasing nitric oxide. Nitric oxide is known to be inhibitory within the PVN and is also co-localized with NMDA NR1 receptors in the PVN (Li et al., 2001). It may well be that CNP elicits inhibition of the glutamatergic neuron in the PVN *via* a nitric oxide mechanism. However, the full details of such a mechanism for CNP in the PVN remains to be examined (Figure 10).

There is growing evidence to support the involvement of CNP in cardiovascular diseases (Lee et al., 2015; Sangaralingham et al., 2015). However, the biological role of CNP in conditions such as heart failure has yet to be as clearly defined. It has been reported that in patients with chronic heart failure, the heart produces CNP (Kalra et al., 2003). A selective increase of the NPR-C has also been documented in the hearts of patients with heart failure (Kuhn et al., 2004). In the ovine model of heart failure, a substantial decrease of natriuretic peptides was found in the hypothalamus (Pemberton et al., 2002). However, the physiological and pathological role of natriuretic peptides in the hypothalamus remains to be examined. Among the various natriuretic peptide components, CNP appears to be the most involved and likely candidate as a neuromodulator in the central nervous system (Herman et al., 1993; Kaneko et al., 1993). CNP signaling *via* the NPR-B has been shown to be sympatho-inhibitory *via* a direct action on stellate sympathetic neurons (Buttgereit et al., 2016). It is of interest to note that BNP is regarded as an important biomarker for the diagnosis, risk stratification, and prediction of death in patients with chronic heart failure (Anand et al., 2003; Braunwald 2008). Overall, natriuretic peptides are promising candidates for treating congestive heart failure because of their wide range of beneficial effects on the cardiovascular system (Levin et al., 1998; Mills et al., 1999; Colucci et al., 2000). Natriuretic peptides have been used in the clinical setting for the treatment of heart failure for some time now, and treatment approaches that enhance their effects are now starting to be used in the treatment of chronic heart failure. In light of the observations in this study that CNP is intimately involved in regulation of the volume reflex and thus fluid balance, it certainly deserves further investigation in the future to clarify the role of CNP in the CNS in disease conditions, such as chronic heart failure and hypertension characterized by augmented sympathetic drive and altered fluid balance (Leenen 2007; Floras 2009).

## Perspectives

Our results describe a novel and specific role for CNP in the PVN for the regulation of RSNA and thus renal function. These findings show a specific inhibitory role for the CNP within the PVN for the regulation of RSNA. These results provide new insights into the central regulation of RSNA during the volume reflex and provide potential targets for manipulating fluid balance in normal and disease conditions such as hypertension and heart failure, involving the alterations of the cardio-renal system.

## Data Availability

The original contributions presented in the study are included in the article/supplementary material, further inquiries can be directed to the corresponding author.
